# 2-(2-Quinol­yl)quinolinium nitrate

**DOI:** 10.1107/S1600536808012579

**Published:** 2008-05-03

**Authors:** Anita Abedi, Arezoo Bahrami Shabestari, Vahid Amani

**Affiliations:** a Department of Chemistry, Islamic Azad University, North Tehran Branch, Tehran, Iran; b Department of Chemistry, Islamic Azad University, Shahr-e-Rey Branch, Tehran, Iran

## Abstract

In the cation of the title compound, C_18_H_13_N_2_^+^·NO_3_^−^, the two bicyclic ring systems form a dihedral angle of 3.84 (4)°. The nitrate anion is disordered over two orientations in a 0.9:0.1 ratio. In the crystal structure, the cations form stacks along the *a* axis, with short inter­molecular contacts [C⋯C = 3.330 (3) and 3.345 (4) Å], and link to the anions *via* N—H⋯O hydrogen bonds.

## Related literature

For related literature, see: Smith *et al.* (1999 ▶); Zafar *et al.* (2000 ▶); Rafizadeh *et al.* (2006 ▶); Yousefi *et al.* (2007 ▶); Parlow & Hartl (1979 ▶).
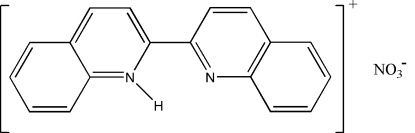


## Experimental

### 

#### Crystal data

C_18_H_13_N_2_^+^·NO_3_^−^
*M* *_r_* = 319.31
Monoclinic, 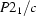

*a* = 6.9756 (6) Å
*b* = 10.6408 (9) Å
*c* = 19.1226 (15) Å
β = 94.399 (2)°
*V* = 1415.2 (2) Å^3^
*Z* = 4
Mo *K*α radiation
μ = 0.10 mm^−1^
*T* = 120 (2) K
0.45 × 0.30 × 0.25 mm

#### Data collection

Bruker SMART 1000 CCD area-detector diffractometer
Absorption correction: none
15078 measured reflections
3739 independent reflections
2115 reflections with *I* > 2σ(*I*)
*R*_int_ = 0.038

#### Refinement

*R*[*F* ^2^ > 2σ(*F* ^2^)] = 0.057
*wR*(*F* ^2^) = 0.136
*S* = 0.97
3739 reflections
229 parameters
3 restraints
H-atom parameters constrained
Δρ_max_ = 0.31 e Å^−3^
Δρ_min_ = −0.29 e Å^−3^

### 

Data collection: *SMART* (Bruker, 1998 ▶); cell refinement: *SAINT-Plus* (Bruker, 1998 ▶); data reduction: *SAINT-Plus*; program(s) used to solve structure: *SHELXTL* (Sheldrick, 2008 ▶); program(s) used to refine structure: *SHELXTL*; molecular graphics: *SHELXTL*; software used to prepare material for publication: *SHELXTL*.

## Supplementary Material

Crystal structure: contains datablocks I, global. DOI: 10.1107/S1600536808012579/cv2401sup1.cif

Structure factors: contains datablocks I. DOI: 10.1107/S1600536808012579/cv2401Isup2.hkl

Additional supplementary materials:  crystallographic information; 3D view; checkCIF report

## Figures and Tables

**Table 1:** Hydrogen-bond geometry (Å, °)

*D*—H⋯*A*	*D*—H	H⋯*A*	*D*⋯*A*	*D*—H⋯*A*
N2—H2N⋯O1	0.91	1.92	2.766 (2)	153

**Fig. 1.**
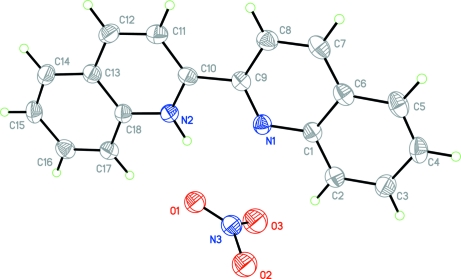
The content of asymmetric unit of (I) showing the atomic numbering and 50% probability displacement ellipsoids. Only major part of the disordered nitrate anion is shown.

## supplementary crystallographic information

### Comment

In recent years, there has been considerable interest in proton transfer systems and their structures (Smith *et al.*, 1999; Zafar *et al.*, 2000; Rafizadeh *et al.*, 2006; Yousefi *et al.*, 2007). To our knowledge, there is only one proton-transfer system with 2,2'-biquinolinyl, such as [(biq.H)(I_2_Cl_3_)] [biq.H = 2-(2-quinolinyl)quinolinium], which was structurally characterized (Parlow & Hartl, 1979). Herewith we report the synthesis and crystal structure of the title compound, (I).

The asymmetric unit of (I) contains one cation and one anion (Fig. 1). In the cation, two bicycles form a dihedral angle of 3.84 (4)%. In the crystal structure, the cations form stacks along the *a* axis with short intermolecular contacts [C···C = 3.330 (3) and 3.345 (4)Å] linking the anions *via* N—H···O hydrogen bonds.

### Experimental

For the preparation of the title compound, (I), a solution of 2,2'-biquinolinyl (0.20 g, 0.78 mmol) in HNO_3_ 0.5 *M* (10 ml) was added to a solution of La(NO_3_)_3_.6H_2_O, (0.11 g, 0.26 mmol) in water (5 ml) and the resulting yellow solution was stirred at 333 K for 2 h. Then, it was left to evaporate slowly at room temperature. The suitable crystals for X-ray diffraction experiment were obtained by methanol diffusion in a solution of yellow precipitated in DMSO after one week (yield 0.19 g, 76.2%, m.p 496–497 K).

### Refinement

C-bound H atoms were geometrically positioned (C-H 0.95 Å). The H atom of NH group was located on a difference Fourier map, but placed in idealized position (N-H 0.91 Å). All H atoms were refined in riding model approximation, with *U*_iso_(H) = 1.2 U_eq_ of the parent atom. The NO_3_ anion was treated as disordered between two orientations with the occupancies fixed to 0.9 and 0.1, respectively.

### Crystal data

**Table:**

C_18_H_13_N_2_^+^·NO_3_^–^	*F*_000_ = 664
*M**_r_* = 319.31	*D*_x_ = 1.499 Mg m^−^^3^
Monoclinic, *P*2_1_/*c*	Mo *K*α radiation λ = 0.71073 Å
Hall symbol: -P 2ybc	Cell parameters from 937 reflections
*a* = 6.9756 (6) Å	θ = 3–29º
*b* = 10.6408 (9) Å	µ = 0.11 mm^−^^1^
*c* = 19.1226 (15) Å	*T* = 120 (2) K
β = 94.399 (2)º	Block, yellow
*V* = 1415.2 (2) Å^3^	0.45 × 0.30 × 0.25 mm
*Z* = 4	

### Data collection

**Table:**

Bruker SMART 1000 CCD area-detector diffractometer	2115 reflections with *I* > 2σ(*I*)
Radiation source: normal-focus sealed tube	*R*_int_ = 0.038
Monochromator: graphite	θ_max_ = 29.0º
*T* = 120(2) K	θ_min_ = 2.1º
ω scans	*h* = −9→9
Absorption correction: none	*k* = −14→14
15078 measured reflections	*l* = −25→26
3739 independent reflections	

### Refinement

**Table:**

Refinement on *F*^2^	Secondary atom site location: difference Fourier map
Least-squares matrix: full	Hydrogen site location: mixed
*R*[*F*^2^ > 2σ(*F*^2^)] = 0.057	H-atom parameters constrained
*wR*(*F*^2^) = 0.136	*w* = 1/[σ^2^(*F*_o_^2^) + (0.0551*P*)^2^ + 0.46*P*] where *P* = (*F*_o_^2^ + 2*F*_c_^2^)/3
*S* = 0.97	(Δ/σ)_max_ < 0.001
3739 reflections	Δρ_max_ = 0.31 e Å^−^^3^
229 parameters	Δρ_min_ = −0.29 e Å^−^^3^
3 restraints	Extinction correction: none
Primary atom site location: structure-invariant direct methods	

### Special details

**Table:**

Geometry. All e.s.d.'s (except the e.s.d. in the dihedral angle between two l.s. planes) are estimated using the full covariance matrix. The cell e.s.d.'s are taken into account individually in the estimation of e.s.d.'s in distances, angles and torsion angles; correlations between e.s.d.'s in cell parameters are only used when they are defined by crystal symmetry. An approximate (isotropic) treatment of cell e.s.d.'s is used for estimating e.s.d.'s involving l.s. planes.
Refinement. Refinement of *F*^2^ against ALL reflections. The weighted *R*-factor *wR* and goodness of fit *S* are based on *F*^2^, conventional *R*-factors *R* are based on *F*, with *F* set to zero for negative *F*^2^. The threshold expression of *F*^2^ > σ(*F*^2^) is used only for calculating *R*-factors(gt) *etc*. and is not relevant to the choice of reflections for refinement. *R*-factors based on *F*^2^ are statistically about twice as large as those based on *F*, and *R*- factors based on ALL data will be even larger.

### Fractional atomic coordinates and isotropic or equivalent isotropic displacement parameters (Å^2^)

**Table:**

	*x*	*y*	*z*	*U*_iso_*/*U*_eq_	Occ. (<1)
N1	0.2846 (2)	0.12303 (14)	0.47447 (7)	0.0261 (3)	
N2	0.21783 (19)	−0.05200 (13)	0.56913 (7)	0.0245 (3)	
H2N	0.2249	0.0316	0.5802	0.029*	
C1	0.3241 (2)	0.21681 (17)	0.42885 (9)	0.0265 (4)	
C2	0.3386 (3)	0.34167 (17)	0.45393 (10)	0.0320 (4)	
H2A	0.3180	0.3593	0.5015	0.038*	
C3	0.3821 (3)	0.43694 (19)	0.41010 (10)	0.0352 (5)	
H3A	0.3920	0.5205	0.4274	0.042*	
C4	0.4124 (3)	0.4124 (2)	0.33902 (10)	0.0347 (5)	
H4A	0.4448	0.4794	0.3093	0.042*	
C5	0.3955 (3)	0.29307 (19)	0.31289 (10)	0.0329 (4)	
H5A	0.4127	0.2780	0.2648	0.040*	
C6	0.3524 (2)	0.19166 (18)	0.35718 (9)	0.0283 (4)	
C7	0.3393 (3)	0.06604 (19)	0.33471 (9)	0.0319 (4)	
H7A	0.3553	0.0460	0.2871	0.038*	
C8	0.3037 (3)	−0.02721 (18)	0.38088 (9)	0.0307 (4)	
H8A	0.2981	−0.1126	0.3663	0.037*	
C9	0.2754 (2)	0.00614 (17)	0.45085 (9)	0.0243 (4)	
C10	0.2313 (2)	−0.09095 (17)	0.50285 (9)	0.0249 (4)	
C11	0.2028 (3)	−0.21806 (17)	0.48705 (10)	0.0309 (4)	
H11A	0.2123	−0.2471	0.4405	0.037*	
C12	0.1613 (3)	−0.30107 (17)	0.53859 (10)	0.0321 (4)	
H12A	0.1413	−0.3873	0.5273	0.038*	
C13	0.1480 (2)	−0.26001 (17)	0.60813 (9)	0.0272 (4)	
C14	0.1044 (2)	−0.34060 (17)	0.66293 (10)	0.0313 (4)	
H14A	0.0826	−0.4274	0.6539	0.038*	
C15	0.0931 (3)	−0.29430 (18)	0.72935 (10)	0.0332 (4)	
H15A	0.0621	−0.3492	0.7660	0.040*	
C16	0.1272 (3)	−0.16592 (18)	0.74375 (10)	0.0326 (4)	
H16A	0.1207	−0.1355	0.7902	0.039*	
C17	0.1696 (2)	−0.08437 (17)	0.69156 (9)	0.0285 (4)	
H17A	0.1928	0.0020	0.7015	0.034*	
C18	0.1781 (2)	−0.13100 (17)	0.62339 (9)	0.0248 (4)	
N3	0.2376 (3)	0.27946 (17)	0.62588 (8)	0.0385 (4)	
O1	0.1506 (3)	0.17769 (16)	0.63037 (10)	0.0433 (5)	0.90
O2	0.1410 (3)	0.37683 (15)	0.60925 (9)	0.0502 (5)	0.90
O3	0.4157 (3)	0.2874 (2)	0.63555 (9)	0.0555 (5)	0.90
O1'	0.0850 (18)	0.2168 (18)	0.6230 (13)	0.060 (7)*	0.10
O2'	0.264 (3)	0.3897 (8)	0.6450 (11)	0.077 (6)*	0.10
O3'	0.3749 (19)	0.2024 (14)	0.6346 (9)	0.054 (4)*	0.10

### Atomic displacement parameters (Å^2^)

**Table:**

	*U*^11^	*U*^22^	*U*^33^	*U*^12^	*U*^13^	*U*^23^
N1	0.0253 (8)	0.0271 (8)	0.0258 (8)	0.0003 (6)	0.0022 (6)	0.0036 (6)
N2	0.0255 (8)	0.0229 (7)	0.0249 (8)	0.0000 (6)	0.0011 (6)	−0.0003 (6)
C1	0.0239 (9)	0.0306 (10)	0.0249 (9)	0.0021 (7)	0.0017 (7)	0.0031 (8)
C2	0.0371 (11)	0.0314 (10)	0.0280 (10)	0.0010 (8)	0.0063 (8)	0.0017 (8)
C3	0.0368 (11)	0.0306 (10)	0.0384 (11)	0.0008 (8)	0.0044 (9)	0.0054 (8)
C4	0.0289 (10)	0.0431 (12)	0.0323 (10)	0.0004 (8)	0.0027 (8)	0.0144 (9)
C5	0.0274 (10)	0.0473 (12)	0.0239 (9)	0.0000 (9)	0.0010 (7)	0.0069 (8)
C6	0.0214 (9)	0.0393 (11)	0.0243 (9)	0.0021 (8)	0.0014 (7)	0.0027 (8)
C7	0.0286 (10)	0.0442 (12)	0.0230 (9)	−0.0017 (8)	0.0035 (7)	−0.0013 (8)
C8	0.0329 (10)	0.0318 (10)	0.0273 (10)	−0.0010 (8)	0.0029 (8)	−0.0046 (8)
C9	0.0214 (8)	0.0288 (9)	0.0226 (8)	0.0006 (7)	0.0001 (7)	−0.0005 (7)
C10	0.0203 (8)	0.0292 (10)	0.0251 (9)	0.0016 (7)	0.0013 (7)	−0.0017 (7)
C11	0.0332 (10)	0.0299 (10)	0.0295 (10)	0.0002 (8)	0.0026 (8)	−0.0047 (8)
C12	0.0310 (10)	0.0240 (9)	0.0412 (11)	−0.0010 (8)	0.0029 (8)	−0.0041 (8)
C13	0.0215 (9)	0.0282 (9)	0.0319 (10)	0.0015 (7)	0.0027 (7)	0.0018 (8)
C14	0.0280 (10)	0.0241 (9)	0.0417 (11)	−0.0014 (7)	0.0024 (8)	0.0046 (8)
C15	0.0290 (10)	0.0336 (10)	0.0375 (11)	−0.0009 (8)	0.0053 (8)	0.0118 (8)
C16	0.0339 (10)	0.0364 (11)	0.0278 (10)	0.0036 (8)	0.0048 (8)	0.0024 (8)
C17	0.0287 (10)	0.0288 (9)	0.0282 (9)	0.0013 (8)	0.0027 (7)	−0.0003 (8)
C18	0.0203 (8)	0.0258 (9)	0.0283 (9)	0.0016 (7)	0.0017 (7)	0.0036 (7)
N3	0.0534 (11)	0.0360 (10)	0.0272 (9)	−0.0011 (9)	0.0102 (8)	−0.0040 (7)
O1	0.0697 (12)	0.0249 (9)	0.0380 (10)	−0.0089 (10)	0.0211 (9)	−0.0020 (8)
O2	0.0790 (13)	0.0276 (9)	0.0460 (10)	0.0114 (9)	0.0186 (9)	0.0005 (7)
O3	0.0514 (12)	0.0705 (14)	0.0440 (11)	−0.0145 (10)	0.0011 (8)	0.0099 (9)

### Geometric parameters (Å, °)

**Table:**

N1—C9	1.323 (2)	C10—C11	1.397 (3)
N1—C1	1.367 (2)	C11—C12	1.371 (3)
N2—C10	1.344 (2)	C11—H11A	0.9500
N2—C18	1.380 (2)	C12—C13	1.410 (3)
N2—H2N	0.910	C12—H12A	0.9500
C1—C2	1.413 (3)	C13—C14	1.405 (2)
C1—C6	1.425 (2)	C13—C18	1.416 (3)
C2—C3	1.364 (3)	C14—C15	1.370 (3)
C2—H2A	0.9500	C14—H14A	0.9500
C3—C4	1.416 (3)	C15—C16	1.410 (3)
C3—H3A	0.9500	C15—H15A	0.9500
C4—C5	1.366 (3)	C16—C17	1.372 (3)
C4—H4A	0.9500	C16—H16A	0.9500
C5—C6	1.418 (3)	C17—C18	1.400 (2)
C5—H5A	0.9500	C17—H17A	0.9500
C6—C7	1.405 (3)	N3—O2'	1.239 (5)
C7—C8	1.364 (3)	N3—O3	1.245 (2)
C7—H7A	0.9500	N3—O1	1.247 (2)
C8—C9	1.413 (2)	N3—O1'	1.253 (5)
C8—H8A	0.9500	N3—O3'	1.263 (5)
C9—C10	1.483 (2)	N3—O2	1.263 (2)
			
N1···C18^i^	3.606 (3)	C6···C14^i^	3.550 (4)
N1···C10^ii^	3.388 (3)	C18···C6^ii^	3.330 (3)
C1···C13^i^	3.345 (4)		
			
C9—N1—C1	118.36 (15)	N2—C10—C9	116.81 (15)
C10—N2—C18	123.64 (15)	C11—C10—C9	124.27 (16)
C10—N2—H2N	120.8	C12—C11—C10	120.19 (17)
C18—N2—H2N	115.4	C12—C11—H11A	119.9
N1—C1—C2	118.83 (16)	C10—C11—H11A	119.9
N1—C1—C6	121.75 (16)	C11—C12—C13	120.75 (17)
C2—C1—C6	119.42 (16)	C11—C12—H12A	119.6
C3—C2—C1	120.24 (17)	C13—C12—H12A	119.6
C3—C2—H2A	119.9	C14—C13—C12	123.24 (17)
C1—C2—H2A	119.9	C14—C13—C18	118.36 (17)
C2—C3—C4	120.60 (19)	C12—C13—C18	118.40 (16)
C2—C3—H3A	119.7	C15—C14—C13	120.20 (17)
C4—C3—H3A	119.7	C15—C14—H14A	119.9
C5—C4—C3	120.51 (18)	C13—C14—H14A	119.9
C5—C4—H4A	119.7	C14—C15—C16	120.56 (17)
C3—C4—H4A	119.7	C14—C15—H15A	119.7
C4—C5—C6	120.33 (17)	C16—C15—H15A	119.7
C4—C5—H5A	119.8	C17—C16—C15	120.89 (18)
C6—C5—H5A	119.8	C17—C16—H16A	119.6
C7—C6—C5	123.57 (17)	C15—C16—H16A	119.6
C7—C6—C1	117.54 (16)	C16—C17—C18	118.69 (17)
C5—C6—C1	118.88 (17)	C16—C17—H17A	120.7
C8—C7—C6	120.34 (17)	C18—C17—H17A	120.7
C8—C7—H7A	119.8	N2—C18—C17	120.62 (16)
C6—C7—H7A	119.8	N2—C18—C13	118.09 (16)
C7—C8—C9	118.43 (17)	C17—C18—C13	121.29 (16)
C7—C8—H8A	120.8	O3—N3—O1	122.3 (2)
C9—C8—H8A	120.8	O2'—N3—O1'	128.7 (14)
N1—C9—C8	123.57 (16)	O2'—N3—O3'	118.7 (14)
N1—C9—C10	115.67 (15)	O1'—N3—O3'	107.1 (13)
C8—C9—C10	120.77 (16)	O3—N3—O2	119.2 (2)
N2—C10—C11	118.92 (16)	O1—N3—O2	118.5 (2)
			
C9—N1—C1—C2	−178.56 (16)	N1—C9—C10—N2	−4.2 (2)
C9—N1—C1—C6	0.9 (2)	C8—C9—C10—N2	176.18 (15)
N1—C1—C2—C3	178.55 (17)	N1—C9—C10—C11	175.52 (16)
C6—C1—C2—C3	−1.0 (3)	C8—C9—C10—C11	−4.1 (3)
C1—C2—C3—C4	0.3 (3)	N2—C10—C11—C12	0.2 (3)
C2—C3—C4—C5	1.1 (3)	C9—C10—C11—C12	−179.57 (16)
C3—C4—C5—C6	−1.7 (3)	C10—C11—C12—C13	−0.4 (3)
C4—C5—C6—C7	−177.72 (18)	C11—C12—C13—C14	179.46 (17)
C4—C5—C6—C1	1.0 (3)	C11—C12—C13—C18	0.2 (3)
N1—C1—C6—C7	−0.3 (3)	C12—C13—C14—C15	−179.80 (17)
C2—C1—C6—C7	179.14 (16)	C18—C13—C14—C15	−0.5 (3)
N1—C1—C6—C5	−179.15 (16)	C13—C14—C15—C16	−0.7 (3)
C2—C1—C6—C5	0.3 (3)	C14—C15—C16—C17	0.9 (3)
C5—C6—C7—C8	177.74 (17)	C15—C16—C17—C18	0.1 (3)
C1—C6—C7—C8	−1.0 (3)	C10—N2—C18—C17	178.81 (15)
C6—C7—C8—C9	1.7 (3)	C10—N2—C18—C13	−0.6 (2)
C1—N1—C9—C8	−0.2 (2)	C16—C17—C18—N2	179.18 (16)
C1—N1—C9—C10	−179.75 (15)	C16—C17—C18—C13	−1.4 (3)
C7—C8—C9—N1	−1.1 (3)	C14—C13—C18—N2	−178.98 (15)
C7—C8—C9—C10	178.41 (16)	C12—C13—C18—N2	0.3 (2)
C18—N2—C10—C11	0.4 (2)	C14—C13—C18—C17	1.6 (2)
C18—N2—C10—C9	−179.86 (14)	C12—C13—C18—C17	−179.08 (16)

Symmetry codes: (i) −*x*, −*y*, −*z*+1; (ii) −*x*+1, −*y*, −*z*+1.

### Hydrogen-bond geometry (Å, °)

**Table:**

*D*—H···*A*	*D*—H	H···*A*	*D*···*A*	*D*—H···*A*
N2—H2N···O1	0.91	1.92	2.766 (2)	153
